# Exploring the role of pancreatic enzyme replacement therapy in patients with short bowel syndrome: Nutritional and Anthropometric Outcomes

**DOI:** 10.1016/j.intf.2025.100049

**Published:** 2025-03-24

**Authors:** Sirine Belaid, Vikram Raghu, Feras Alissa, Jeffrey Rudolph

**Affiliations:** Division of Pediatric Gastroenterology, Hepatology and Nutrition, University of Pittsburgh Medical Center Children’s Hospital of Pittsburgh, Pittsburgh, PA, USA

**Keywords:** Short bowel syndrome (SBS), Pancreatic enzyme replacement therapy (PERT), Pancreatic insufficiency (PI)

## Abstract

**Background:**

Pancreatic enzyme replacement therapy (PERT) has been proposed as a treatment for patients with short bowel syndrome (SBS) to address fat malabsorption and promote growth. However, research on the effectiveness of PERT in this population remains limited. This study aims to investigate the impact of PERT on nutritional status and anthropometric measurements in individuals with SBS.

**Material and Methods:**

We retrospectively analyzed medical records of 14 patients with SBS under 25 years at our institution who were prescribed PERT over a twelve-year period. Nutritional status factors including enteral and parenteral calorie intake, fat-soluble vitamin deficiency, vitamin supplement use, along with anthropometric measurements, were assessed across three-time frames: 6 months pre-PERT administration, at PERT initiation, and 6 months post-PERT. Statistical tests, including Mann-Whitney rank sum, Chi-squared, and regression tests, were conducted with a significance level set at 0.05.

**Results:**

Of the 14 patients with SBS who were using PERT, six had elastase levels suggesting pancreatic insufficiency (PI). Among the 14 patients, one discontinued parenteral nutrition (PN) support after 6 months of PERT. Fat soluble vitamin deficiency persisted among three patients despite vitamin supplementation. Six months post-PERT, there was a non-significant decrease in all mean anthropometric z-scores, except for the weight z-scores (p > 0.84). Significant decreases in mean BMI z-scores (p = 0.03) and non-significant reductions in all remaining anthropometric measurements (p > 0.13) were observed among patients with PI following 6 months of PERT use.

**Conclusion:**

PERT utilization may not significantly address fat malabsorption or promote growth in patients with SBS, likely due to anatomical constraints of a shortened bowel that affect nutrient absorption. Further investigation is warranted to determine the efficacy of PERT as a treatment tool for patients with SBS.

## Introduction

Intestinal failure (IF) secondary to bowel resection diminishes gut surface area for nutrient absorption which in turn increases the risk of macronutrient malabsorption, micronutrient malabsorption and failure to thrive [1], [2]. Literature suggests that patients with short bowel syndrome (SBS) are prone to inadequate pancreatic enzyme secretion [3], [4]. Desynchronization between duodenal hormonal secretion and passage of nutrients in the gut may cause inadequate pancreatic enzyme secretion which can lead to macronutrient maldigestion and fat-soluble vitamin deficiencies [3], [4], [5], [6]. Additional research is required to better understand the association between suboptimal pancreatic enzyme secretion and SBS.

Studies propose that pancreatic enzyme replacement therapy (PERT) may be beneficial for patients with SBS who experience fat maldigestion and malabsorption, which can subsequently affect growth and development [3], [4], [5]. However, further investigation is warranted to study the utilization of PERT among this population. The purpose of this study is to explore the use of PERT on nutritional status and anthropometric measurements among patients with SBS.

## Material and methods

### Study design and inclusion/exclusion criteria

We conducted a retrospective observational study to examine the use of PERT among patients with SBS and its effects on nutritional status and growth, measured through caloric intake and anthropometric measurements before and after PERT initiation. Patients were included if they had been seen at the Intestinal Care and Rehabilitation (ICARE) Clinic, had a diagnosis of SBS, were aged between 0 and 25 years at the time of their diagnosis, and had received a PERT prescription between January 2011 and October 2023. Patients were excluded if they did not have SBS, had a diagnosis of cystic fibrosis (CF), or if documentation confirmed they never actually received PERT despite having a prescription. The study was approved by the IRB (#23030125).

### Participants and clinical characteristics

Demographic data were obtained from chart review, and focused on age, gender, race, and ethnicity. Additional data collected from medical records assessed etiology of SBS, age at the time of diagnosis, percent length of bowel remaining and percent of patients with bowel in continuity. Other factors evaluated included number of pancreatitis episodes, change in pancreatic imaging findings, as well as the presence of autoimmune disease, dysmotility disorder, genetic disorder predisposing to pancreatic diseases, hypertriglyceridemia, and additional health conditions.

### Assessment of nutritional status and therapeutic interventions

We studied the nutritional status among our patient population by assessing the caloric intake from enteral nutrition and parenteral nutrition (PN), type of lipid emulsion used, presence of fat-soluble vitamin deficiency and use of vitamin supplements, using INR as a proxy for vitamin K levels. We also looked at the type of PERT prescribed (Pancrelipase like CreonⓇ or ViokaceⓇ, immobilized lipase like RelizorbⓇ), its dose and duration, fecal elastase levels, and use of teduglutide. Teduglutide is a glucagon-like peptide 2 analog commonly used in patients with SBS. It helps reduce gastric secretions, slows gastric emptying and intestinal transit, increases intestinal blood flow, stimulates intestinal growth, and enhances nutrient absorption [7], [8].

### Assessment of anthropometric measurements and statistical analysis

**Fig. 1:**
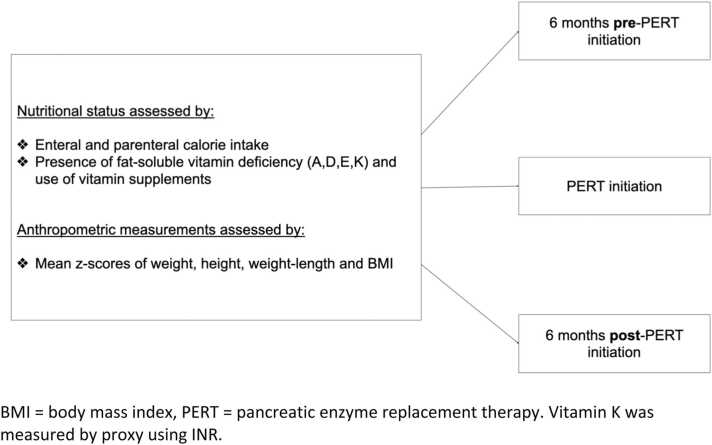
Main outcomes of interest and the study timeframe for our cohort.BMI = body mass index, PERT = pancreatic enzyme replacement therapy. Vitamin K was measured by proxy using INR.

We gathered anthropometric measurements (weight (kg), height (cm), weight-for-length (kg/cm), and Body Mass Index (BMI) (kg/m2)), their percentiles and z-scores) to assess the effect of PERT use on growth. Data collected was divided into 3 main time frames based on the use of PERT - six months prior to PERT use, at time of PERT initiation, and six months post-PERT initiation (Fig. 1). We also repeated the analysis focusing solely on prepubertal and pubescent children to better assess the effect of PERT on growth. The Mann-Whitney rank sum and Chi-squared tests were used to analyze ordinal and categorical variables respectively, and regression tests were utilized for continuous variable analysis. All statistical tests used a minimum alpha level of 0.05 to establish significant results.

## Results

We included 14 patients who met the diagnostic criteria for SBS, were using PERT during the study timeframe, and did not have CF.

### Demographics

Race and ethnicity data from medical records were incomplete for the majority of our patients. The mean age at PERT initiation for our 14 patients was 7.9 years old, with a range from 1 to 22 years. All patients were diagnosed with SBS shortly after birth. Moreover, 57 % of our cohort were males.

### Etiology of SBS

The most common etiologies of SBS were necrotizing enterocolitis (NEC) (n = 4), followed closely by volvulus (n = 3), gastroschisis (n = 3) and intestinal atresia (n = 2) (Table 1). This included one patient who had both intestinal atresia and volvulus. The remaining patients had diagnoses of Hirschsprung disease (n = 1), imperforate anus requiring partial colectomy, and intestinal ganglioneuroma requiring a debulking procedure. The mean percentage of bowel remaining was 25 % (SD: 24.3), and 71 % of patients maintained bowel continuity.

**Table 1:** Characteristics of all 14 subjects in our cohort.

Characteristics	All patients, n = 14
Age at time of SBS diagnosis, mean, years	< 1
Sex, % male	57
Etiologies of SBS	
NEC, n	4
Volvulus, n	3
Gastroschisis, n	3
Intestinal atresia, n	2
Patients with history of bowel resection, n	14
Patients with bowel in continuity, n (%)	10 (71.4)
Remaining % of expected bowel length for age, mean + SD *	25.2 ± 24.3
Small bowel transplant recipient, n	1
Age at time of PERT initiation, mean + SD, years	7.9 + 6.3
PERT type used	
Creon pancrelipase, n	14
Viokace pancrelipase, n	2
Relizorb immobilized lipase, n	0
Duration of PERT	
< 6 months, n	4
> =6 months, n	10
Initial dose of PERT, lipase units/kg	450–900
Patients prescribed PN and/or lipid at time of PERT initiation, n	8
PN and/or lipid kilocalories/kg at time of PERT initation, mean + SD, kcal/kg/day	13.7 ± 16.9
PN and/or lipid kilocalories/kg 6 months post-PERT, mean + SD, kcal/kg/day	11.4 ± 13.0
Enteral kilocalories/kg at time of PERT initiation, mean + SD, kcal/kg/day * *	55.3 ± 43.5
Enteral kilocalories/kg 6 months post-PERT, mean + SD, kcal/kg/day * *	55.2 ± 40.0
Patients on oral diet at time of PERT initiation, n	14
Fat soluble vitamin deficiency at time of PERT initiation	
Vitamin A, n	1
Vitamin D, n	2
Vitamin E, n	2
Vitamin K, n	0
Fat soluble vitamin deficiency 6 months post-PERT	
Vitamin A, n	1
Vitamin D, n	2
Vitamin E, n	2
Vitamin K, n	1
Prescribed vitamin supplements at time of PERT initiation	
Vitamin A, n	0
Vitamin D, n	6
Vitamin E, n	2
Vitamin K, n	0
Prescribed vitamin supplements 6 months post-PERT	
Vitamin A, n	0
Vitamin D, n	8
Vitamin E, n	2
Vitamin K, n	0
Teduglutide use, n	6
Elastase level collected, n	10
Patients with elastase < 200 mcg/g, n (%) ⧫	6 (60)
Weight at time of PERT initiation, mean + SD, kg	21.8 ± 12.8
Weight z-score at time of PERT initiation, mean + SD	−2 ± 1.1
Weight 6 months post-PERT, mean + SD, kg	22.6 ± 12.5
Weight z-score 6 months post-PERT, mean + SD	−2 ± 1.4
Height at time of PERT initiation, mean + SD, cm	112.8 ± 25.3
Height z-score at time of PERT initiation, mean + SD	−2.1 ± 1.3
Height 6 months post-PERT, mean + SD, cm	115.2 ± 24.8
Height z-score 6 months post-PERT, mean + SD	−2.1 ± 1.6
Weight-length at time of PERT initiation, mean + SD, % ↟	42.8 ± 20.8
Weight-length z-score at time of PERT initiation, mean + SD ↟	−0.2 ± 0.6
Weight-length 6 months post-PERT, mean + SD, % ↟	45.3 ± 28.6
Weight length z-score 6 months post-PERT, mean + SD ↟	−0.2 ± 0.9
BMI at time of PERT initiation, mean + SD, %	36.9 ± 23.8
BMI z-score at time of PERT initiation, mean + SD	−0.5 ± 1.0
BMI 6 months post-PERT, mean + SD, %	41.1 ± 28.5
BMI z-score 6 months post-PERT, mean + SD	−0.5 ± 1.4

SBS = short bowel syndrome, NEC = necrotizing enterocolitis, PERT = pancreatic enzyme replacement therapy, PN = parenteral nutrition, BMI = body mass index, *n = 10, * *n = 12, ⧫ n = 10, ↟ n = 11.

### Additional factors studied

Six out of 14 patients were prescribed teduglutide between 0.5- and 5-years following PERT initiation, except for one patient who started teduglutide 2 years prior to PERT initiation. One patient underwent a small bowel transplant and developed gastric carcinoid syndrome with chronic inflammatory arthropathy. No cases of cholestasis, hypertriglyceridemia prior to PERT initiation, or chronic pancreatitis on imaging were found.

Additional conditions observed in our study population included eosinophilic esophagitis (n = 1) and colonic dysmotility requiring diverting surgical loop colostomy (n = 1).

### PERT use and pancreatic insufficiency (PI)

Out of the 14 patients, all used Creon, and two used Viokace for a period of time. For the majority of our patients, the dose of Creon varied between 450 and 900 lipase units/kg body weight per meal. Approximately 29 % (n = 4) discontinued PERT within 6 months of its use. Within our study population, 10 patients had an elastase level collected, and 60 % of them had PI (defined as fecal elastase <200 mcg/g). Improvement in fat malabsorption symptoms varied greatly among our cohort.

### Nutritional status assessment

Six months following PERT use, there was one fewer patient on combined PN and lipid emulsion (decreasing from 8 to 7 patients), with the majority using intralipid emulsion (n = 4) compared to mixed lipid emulsion (n = 1). The mean parenteral calories were reduced by 17 % from the time of PERT initiation to 6 months later, while mean enteral calories remained stable at 55 kcal/kg/day (p < 0.05). Finally, all patients had a general diet at some point during this study timeline. When examining the use of PERT and fat-soluble vitamin deficiency among all 14 patients, we observed that the same two patients who exhibited deficiencies in vitamins D and E at the onset of PERT continued to experience deficiencies 6 months later, despite receiving vitamin D and E supplementation. The individual who had a vitamin A deficiency at the initiation of PERT no longer manifested it after 6 months on PERT therapy, but another patient developed a vitamin A deficiency 6 months post-PERT. Additionally, one patient developed a vitamin K deficiency 6 months after starting PERT. Lastly, we noted an increase in the number of patients on vitamin D supplements (from 6 to 8) 6 months post-PERT compared to the initial period of PERT initiation. There were no patients on vitamin A or K supplements throughout our study period, but two patients remained on vitamin E supplements over the 12-month period.

### Anthropometric measurement assessment

**Fig. 2:**
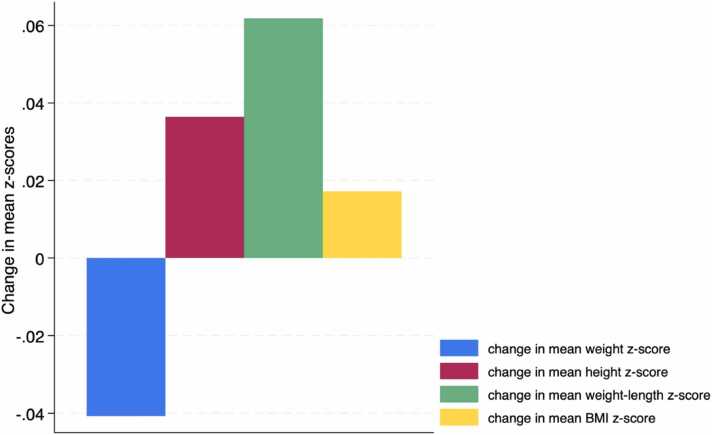
Change in mean z-scores of weight, height, weight-for-length, and BMI at the 6-month mark post-PERT initiation. A non-significant increase was observed in all mean anthropometric z-scores, except for the weight z-scores, after 6 months of PERT use (p > 0.84).

Fig. 2 describes the changes in weight, height, weight-for-length, and BMI z-scores for all 14 patients from the time of PERT initiation to 6 months later. We observed a non-significant increase in the mean z-scores for height, weight-for-length, and BMI after 6 months of PERT, with the exception of the mean weight z-scores (p > 0.84). Furthermore, there was no significant association between changes in any anthropometric z-scores and gender (p > 0.06), age (p > 0.47) or the percentage of bowel remaining (p > 0.15).

**Fig. 3:**
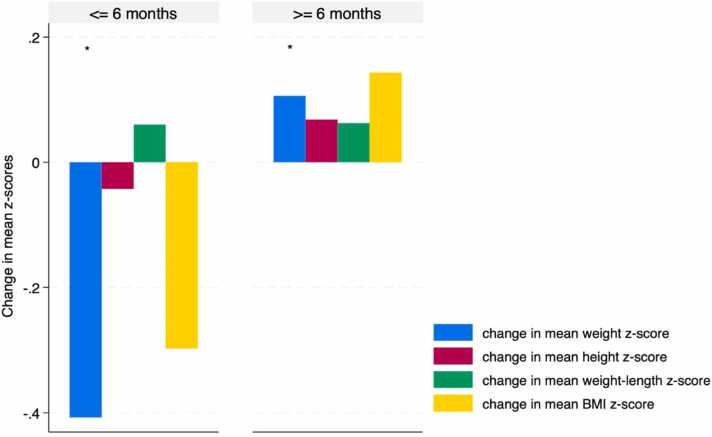
Change in mean anthropometric z-scores following PERT use, stratified by PERT duration. The figure describes a non-significant increase in all anthropometric measurements in patients using PERT for 6 months or more (p > 0.54), except for the weight z-scores (*p = 0.05).

Patients who used PERT for 6 months or more demonstrated a non-significant rise in all anthropometric measurements compared to those who used PERT for less than 6 months (p > 0.54), except for the weight z-scores, which showed a significant change (p = 0.05), as depicted in Fig. 3.

**Fig. 4:**
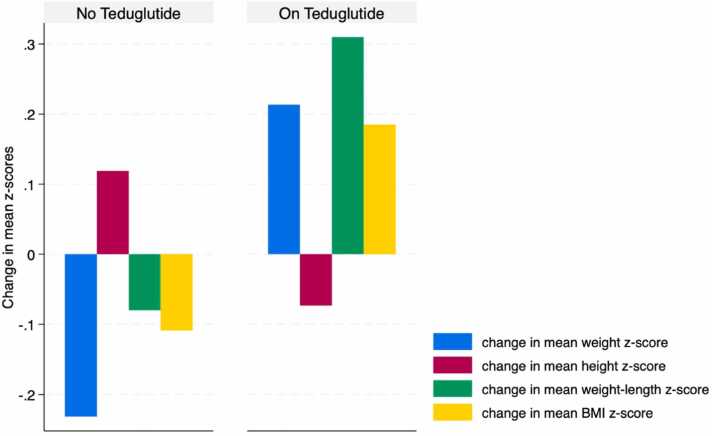
Change in mean anthropometric z-scores following PERT use, stratified by teduglutide use. A non-significant decrease was observed in all mean anthropometric z-scores, except for the height z-scores, after 6 months of PERT use, among those not prescribed teduglutide (p > 0.14).

**Fig. 5:**
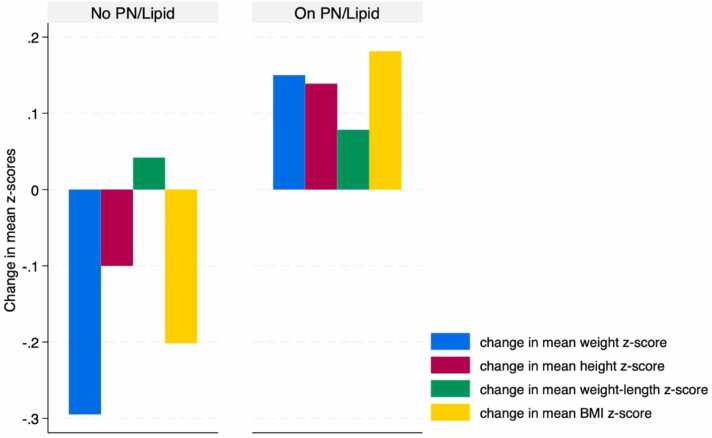
Change in mean anthropometric z-scores after 6 months of PERT use, stratified by PN and/or lipid use at the time of PERT initiation. A non-significant decrease was observed in all mean anthropometric z-scores, except for the weight-for-length z-scores, after 6 months of PERT use, among those not using PN and/or lipid (p > 0.21).

Patients who were not prescribed teduglutide showed a non-significant negative impact on all anthropometric z-scores, except for the height z-scores, 6 months post-PERT (p > 0.14), compared to those using teduglutide, as demonstrated in Fig. 4. Additionally, patients who did not receive PN and/or lipid emulsions at the time of PERT initiation had a non-significant decrease in all anthropometric measurements, except for the weight-for-length z-scores, after 6 months of therapy (p > 0.21), as illustrated in Fig. 5.

**Fig. 6:**
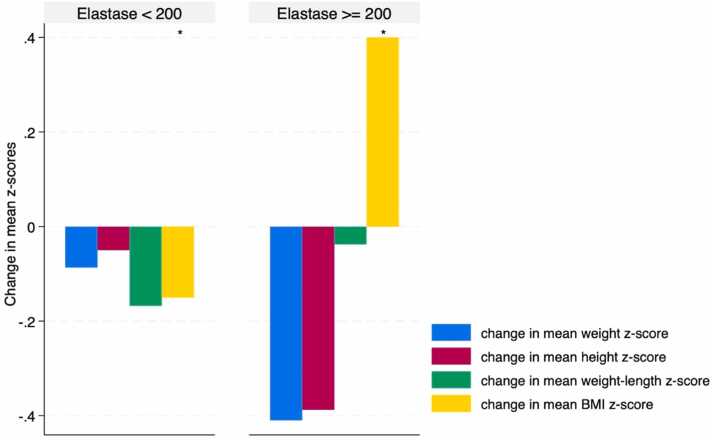
Change in mean anthropometric measurements following 6 months of PERT use, stratified by elastase levels. A non-significant decrease in all anthropometric measurements in patients with PI (elastase level <200 mcg/g) (p > 0.13), except for the BMI z-scores (*p = 0.03), after 6 months of PERT use.

Patients with PI (elastase <200 mcg/g) showed a non-significant decrease in all anthropometric z-scores after 6 months of PERT use (p > 0.13), except for a significant decrease in mean BMI z-scores (p = 0.03), as illustrated in Fig. 6.

We analyzed the 12 prepubertal and pubescent patients, the oldest of whom was 14 years old, to focus exclusively on growth. We found consistent, non-significant anthropometric outcomes 6 months post-PERT, when examining factors such as PERT duration, teduglutide use, PN and/or lipid use at the time of PERT initiation, and elastase levels.

## Discussion

Among the 14 patients with SBS who used PERT, we observed a non-significant reduction in mean parenteral calories after 6 months of PERT use, likely due to multiple factors. We also observed that fat-soluble vitamin deficiencies did not improve with PERT use. In fact, the number of patients with deficiencies in vitamins A, D, and E remained roughly the same, and one additional patient developed a vitamin K deficiency 6 months after starting PERT. These findings are particularly notable given that many patients continued to use vitamin D and E supplements six months post-PERT. This raises the possibility that factors such as poor fat absorption may be contributing to the malabsorption of both fat-soluble vitamins and supplements. Thus, while PERT can assist with fat digestion, it may be insufficient if the gut cannot effectively absorb nutrients and supplements.

When we assessed changes in mean anthropometric z-scores from the time of PERT initiation to six months later, we found a non-significant increase in all measurements, except for weight z-scores. Notably, there was no association between age, gender, or the percentage of bowel remaining and changes in anthropometric measurements following PERT use.

When we examined the role of PERT duration, we found that the rise in anthropometric measurements among the 10 patients who used PERT for six months or more was potentially misleading due to the use of PN and/or lipid emulsion and teduglutide in 8 and 6 patients, respectively, which likely influenced these results (Fig. 3). In contrast, the four patients who used PERT for less than six months (ranging from 1 to 5 months) showed significant weight loss. None of these patients were prescribed teduglutide or received parenteral nutrition, suggesting that PERT alone may not enhance growth outcomes.

We then aimed to individually assess the use of teduglutide and PN and/or lipid in these patients, as both can influence caloric intake and optimize absorption, ultimately impacting weight and linear growth. When excluding teduglutide from consideration, we noted a non-significant decrease in all anthropometric parameters, except for the height z-scores, at the six-month mark (Fig. 4). Among the 8 patients without teduglutide, 50 % used PERT for six months or more, and 25 % used PN and/or lipid. As a next step, we evaluated the impact of PN and/or lipid on these patients (Fig. 5) to clarify the effects of PERT on growth. Among the six patients who did not use any form of parenteral nutrition following the six-month period of PERT use, one-third had used PERT for 6 months or more. We observed a non-significant decrease in all anthropometric measurements, except for the weight-for-length z-scores (Fig. 5). Importantly, these six patients were not prescribed teduglutide. This again suggests that PERT alone, in the absence of teduglutide and PN and/or lipid, may be ineffective in promoting growth.

We specifically examined the impact of PERT in patients with exocrine PI. Of the 10 patients with an elastase level measured, 6 were suspected to have PI, as indicated by a fecal elastase < 200 mcg/g. However, it is important to note that results may be artificially low in cases of high-volume output. We found a decrease in all anthropometric measurements, with the BMI z-score changes being significant among patients with PI (Fig. 6). These results were observed despite 83 % of patients using PERT for 6 months or more, with two-thirds receiving teduglutide and two-thirds using parenteral nutrition. This study is significant as it is the first to evaluate the effects of PERT on nutrition and growth in patients with SBS, considering parenteral nutrition, teduglutide use and presence of PI.

Literature suggests that patients with SBS are predisposed to reduced pancreatic enzyme secretion, which could be explained by several potential mechanisms. One such mechanism is the loss of physiological stimulation from nutrient contact with the duodenum, due to duodenum bypass, duodenectomy, or the loss of hormones secreted by the endocrine L cells in the small intestine and proximal colon [3], [6], [9]. The latter leads to rapid transit through the stomach and duodenum [4], [5], [6], [10] and increased gastric acidity, which inactivates both pancreatic enzymes and bile salts—critical for the absorption of fats and fat-soluble vitamins [5], [10]. Moreover, patients who are solely dependent on total parenteral nutrition may experience intestinal and pancreatic atrophy from absence of intraluminal stimulation by nutrients [11], [12]. This phenomenon has been demonstrated in animal studies; however, further research is needed to confirm these findings in humans. Loperamide, a mu-opioid receptor agonist frequently used as an antidiarrheal agent in patients with SBS, can also reduce pancreatic secretion—particularly trypsin—by as much as 50 %, consistent with the effects of opioids [13].

Given these considerations, one might expect that providing PERT to patients with SBS would enhance nutrient digestion, symptom management, and growth. This effect could be further supported by administering antacid agents to prevent an acidic environment that could inactivate PERT [4], [5].

However, our study results suggest that PERT may be insufficient or ineffective in this patient population. This was also reflected in another study which evaluated PERT effectiveness among 11 patients with SBS and failed to show any statistically significant mean change in coefficients of fat and nitrogen absorption (CFA and CNA respectively) following PERT administration [14]. The duration of PERT does not seem to be a limiting factor in our study, as all 14 patients used PERT for at least one month, with 10 using it for six months or more. Previous studies have reported improvements in malabsorptive symptoms, mean stool fat, fat and protein absorption, and growth parameters within two months of initiating PERT [15], [16], [17]. Our findings may be attributed to suboptimal PERT dosing within our cohort, insufficient use of antacids that could contribute to PERT inactivation, and rapid bowel transit, which may not allow enough time for PERT to take effect. Additionally, most pancreatic enzyme supplements, including Creon, are delayed release preparations and do not activate below a certain pH, which may not be achieved by the time the supplements pass through the duodenum. Furthermore, maldigestion might not be the primary issue; rather, malabsorption could be the main concern due to reduced bowel length and function.

Our study has several limitations. Firstly, the small sample size and the retrospective design may affect the generalizability of our findings. Additionally, some patient chart reviews were incomplete, missing crucial data such as race/ethnicity, bowel remaining after surgery, and weight-for-length measurements. There was no standardization of indications for PERT, nor were there consistent changes in management practices over the study period. We could not control participants who discontinued PERT before the six-month mark, nor could we determine how frequently patients experienced periods of poor adherence. This was also the case for parents of young children who could not swallow capsules and resorted to sprinkling the contents on baby food or milk, which some found challenging. Accurately measuring the exact caloric intake from oral diets was challenging, and data could not be collected. Furthermore, due to limited laboratory tests, we were unable to precisely measure parameters like CFA and CNA, and had to rely on anthropometric measurements as indirect indicators of nutrient absorption. Lastly, fecal elastase may not be the most sensitive test for diagnosing PI, as it can yield false positives due to dilution effects in patients with SBS and chronic diarrhea [18].

## Conclusion

Regardless of whether patients with SBS have insufficient or intact pancreatic enzyme secretion, PERT supplementation alone does not significantly enhance nutrition and growth. Future research should involve larger cohorts to validate our findings and better assess the effectiveness of PERT in this population. Ongoing studies are exploring the use of Relizorb, which consists of lipase cartridges that connect directly with tube feeding sets to predigest fats before they reach the gut [19], [20], [21]. This approach aims to mitigate the impact of rapid bowel transit on Relizorb’s effectiveness [21]. However, if malabsorption is the primary issue, even this intervention may prove insufficient. In such cases, it may be beneficial to consider adding teduglutide and bile salt supplements to PERT to enhance nutrient absorption and support growth.

## Ethical clearance

Not required

## Guarantor of the article

Sirine Belaid, no conflict of interest to disclose

## Financial support

No funding sources to disclose

## Funding

This research did not receive any specific grant from funding agencies in the public, commercial or not-for-profit sectors

## Patients consent

Not applicable

## CRediT authorship contribution statement

**Belaid Sirine:** Writing – review & editing, Writing – original draft, Visualization, Validation, Supervision, Software, Resources, Project administration, Methodology, Investigation, Formal analysis, Data curation, Conceptualization. **Raghu Vikram:** Writing – review & editing, Supervision, Resources, Formal analysis. **Alissa Feras:** Writing – review & editing. **Rudolph Jeffrey:** Writing – review & editing.

## Declaration of Competing Interest

The authors declare that they have no known competing financial interests or personal relationships that could have appeared to influence the work reported in this paper.
